# Regulation of G protein-coupled receptors by palmitoylation and cholesterol

**DOI:** 10.1186/1741-7007-10-27

**Published:** 2012-03-19

**Authors:** Alan D Goddard, Anthony Watts

**Affiliations:** 1 Biomembrane Structure Unit, Department of Biochemistry, University of Oxford, South Parks Rd, Oxford, OX1 3QU, UK

## Abstract

Due to their membrane location, G protein-coupled receptors (GPCRs) are subject to regulation by soluble and integral membrane proteins as well as membrane components, including lipids and sterols. GPCRs also undergo a variety of post-translational modifications, including palmitoylation. A recent article by Zheng *et al*. in *BMC Cell Biology *demonstrates cooperative roles for receptor palmitoylation and cholesterol binding in GPCR dimerization and G protein coupling, underlining the complex regulation of these receptors.

See research article http://www.biomedcentral.com/1471-2121/13/6

## Commentary

G protein-coupled receptors (GPCRs) represent the largest family of integral membrane proteins encoded by the human genome and detect a wide variety of ligands, including peptides, hormones, lipids and nucleotides. They transduce extracellular signals across the membrane to activate intracellular signaling cascades via heterotrimeric G proteins. These consist of a Gα, Gβ and Gγ subunit and the precise combination of these subunits determines the cellular response to receptor activation. Additional non-G protein effectors add layers of complexity and diversity to GPCR signaling cascades. Due to their involvement in a wide variety of physiological processes, GPCRs represent excellent pharmaceutical targets [1] and are the target for approximately 30% of drugs.

GPCRs do not exist in isolation in lipid bilayers, but instead interact with components of the bilayer, such as lipids and sterols, as well as with other GPCRs to form dimers and higher order oligomers. It is likely that, at least in some cases, these dimers are of functional significance and can affect the ligand binding and signaling properties of GPCRs [2]. Much recent effort has been directed to elucidating GPCR crystal structures and a number of the structures obtained seem to indicate the presence of receptor dimers. Intriguingly, the β_2_-adrenergic receptor (β_2_AR) crystalized with cholesterol molecules and a post-translationally added palmitate group from each protomer forming most of the dimer interface [3], suggesting a role for lipids and sterols, in addition to protein-protein interactions, in GPCR dimerization. However, it was not clear whether this accurately represented the conformation of the dimer within native lipid bilayers. A recent study in *BMC Cell Biology *by Zheng *et al*. [4] has revealed a complex interplay between cholesterol, palmitate, receptor dimerization and G protein activation. Their study showed that reducing cholesterol levels or preventing palmitoylation of the μ-opioid receptor (OPRM1) reduced receptor dimerization and Gα association. Additionally, preventing palmitoylation reduced the association of OPRM1 with cholesterol, suggesting a functional complex of receptor, palmitate and cholesterol.

## GPCRs and cholesterol

**Figure 1:**
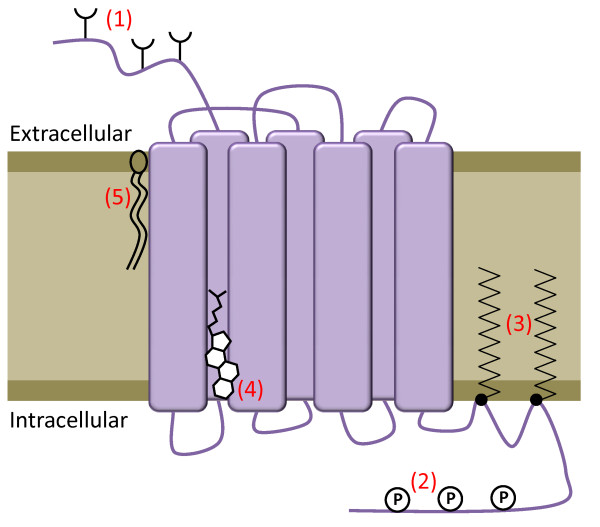
**Schematic of G protein-coupled receptor post-translational modifications and interactions**. GPCRs have seven transmembrane domains (TMs) with an extracellular amino terminus and intracellular carboxyl terminus and alternating intracellular and extracellular loops. They undergo a variety of post-translational modifications and interact with various components of the membrane. (1) The amino terminus and extracellular loops of receptors are often glycosylated, which can be vital for correct cell-surface localization. (2) The carboxy-terminal tail undergoes reversible phosphorylation, which is important in signal desensitization and receptor internalization. (3) Reversible palmitoylation of cysteine residues within intracellular loops and the carboxy-terminal tail results in different loop conformations as the palmitate groups penetrate into the lipid bilayer. (4) Cholesterol can interact at specific sites within the TM helices of GPCRs, in this case TM2 and TM4. (5) GPCRs also may also interact with specific lipid components of the membrane. These interactions and modifications act in concert to modulate the activity of GPCRs.

It has been thought for some time that cholesterol may play an important role or roles in GPCR signaling. Cholesterol is an amphiphilic sterol possessing a small -OH head group and a rigid four-ringed backbone; its size enables it to span approximately half a lipid bilayer, interacting with the hydrophobic tails of phospholipids. Cholesterol can alter the packing of lipids and thus increase membrane order. This has led to the hypothesis that cholesterol is responsible for the formation of lipid rafts and caveolae, regions of the membrane enriched in cholesterol and sphingolipids with higher order than the bulk of the membrane. GPCRs have been found to associate with both regions, and it is believed that this leads to an increased local concentration of receptors and G proteins in the laterally separated regions, increasing the efficiency of signal transduction [5]. In addition to affecting the properties of the membranes in which GPCRs reside, it has been demonstrated that a number of receptors interact directly with cholesterol (Figure 1), which has led to the discovery of consensus cholesterol binding sites in almost half of the family A GPCRs [6]. The importance of cholesterol for GPCR function is supported by that fact that addition of cholesterol or its analogues can greatly increase GPCR stability upon purification and also improve crystal quality. The ability of cholesterol to exert both direct and indirect effects on GPCR activity often complicates interpretation of results, but in a number of cases cholesterol clearly plays an important role in receptor function [6]. However, it should be noted that the effects of cholesterol on a particular receptor are unpredictable, and it has been demonstrated that certain GPCRs can function in *Escherichia coli *membranes, which lack cholesterol [6].

## GPCR palmitoylation

GPCRs are post-translationally modified in a number of ways, including glycosylation, phosphorylation and palmitoylation (Figure 1). Palmitoylation is the addition of palmitic acid (a 16C saturated fatty acid) and occurs on one or more cysteines on the intracellular side of GPCRs. The thioester bond that links the palmitate to the cysteine is cleavable and thus the palmitoylation state of a receptor can be used to regulate its activity. It should be noted that, in rare cases, other lipids can be attached to GPCRs and that palmitoylation can occur on residues other than cysteine [7]. Again, the effects of palmitoylation are unpredictable and GPCR-dependent.

Palmitoylation influences all aspects of GPCR signaling. The palmitoylation state of certain receptors can preferentially direct signaling through particular G proteins and hence give different responses to the same ligand. Up to three palmitate groups can be found on GPCRs and different palmitoylation profiles can result in various conformations of the carboxy-terminal tail, which may select for certain G protein interactions [7]. Palmitoylation can influence the phosphorylation state of the receptor, modulating desensitization and internalization, and can also control internalization independent of phosphorylation. It has been suggested that palmitate binding in the endoplasmic reticulum ensures correct processing and trafficking of receptors, and, once at the cell membrane, may target GPCRs to lipid rafts. However, not all palmitoylated receptors associate with rafts and not all raft-associated GPCRs are palmitoylated [7]. In the case of OPRM1, it appears that palmitoylation and cholesterol association (and presumably raft interactions) are intrinsically linked [4].

## Packed with fat

An intriguing suggestion from the structure of β_2_AR was the ability of cholesterol and palmitate to act in concert to mediate GPCR dimer formation [3]. It is possible that this effect was largely observed due to crystal packing conditions but such an arrangement could influence GPCR activity *in vivo*.

The recent study by Zheng *et al*. [4] systematically investigates the effects of cholesterol and palmitoylation on OPRM1 dimerization and G protein coupling using a variety of colocalization techniques. Cholesterol can be depleted by statins and Zheng *et al*. used the synthetic statin simvastatin to probe the effects of cholesterol on OPRM1. Simvastatin reduced the amount of cholesterol associated with OPRM1 but also decreased receptor dimerization and receptor-G protein interactions. The authors also demonstrate that mutagenesis of the palmitoylated cysteine residue in OPRM1 has no effect on ligand binding but does decrease signaling efficiency, probably by impairing GPCR-G protein association. The same mutant had significantly reduced dimerization, and it was proposed that this was responsible for the reduced G protein coupling. Although it has been suggested that a G protein heterotrimer is too large to signal via a monomeric GPCR, monomeric receptors have been shown to signal efficiently [8], although this ability may be receptor-specific. Finally, Zheng *et al*. demonstrate that the palmitate-free mutant associated more weakly with cholesterol [4], suggesting a functional interaction between the palmitate group and cholesterol.

It is interesting to note that, in contrast to the β_2_AR crystal structure [3], the palmitate group on OPRM1 is not located on the carboxy-terminal tail but instead on the intracellular side of transmembrane domain (TM) 3 [4]. In β_2_AR, the dimer interface involves TM1 and helix 8 (in the carboxy-terminal tail) whereas the OPRM1 dimer interface is predicted to be between TM4 of each protomer, with the palmitate bound to the carboxy-terminal side of TM3. This difference in receptor interface may be driven by the location of palmitoylation and it is possible that regulation of palmitoylation states could dynamically influence the dimerization interfaces of GPCRs. A model of the OPRM1 dimer in which cholesterol and palmitate pack together to facilitate receptor dimerization reveals that cholesterol interactions contribute approximately 25% of the total interaction energy at the homodimer interface [4]. Dimerization of OPRM1 appears to be regulated by palmitate, which recruits cholesterol to stabilize the dimer. Most GPCR dimers are transient, and anything that affects the weak association between protomers may have dramatic effects on the population of dimers at equilibrium.

The complex interplay between receptor, cholesterol and palmitate illustrated by Zheng *et al*. [4] lends support to the model suggested by the crystal structure of β_2_AR [3] by demonstrating a role for sterols and lipids in GPCR dimerization *in vivo*. It remains to be seen if other GPCRs require such modulation to support dimerization. For example, neurotensin receptor 1 can dimerize in artificial lipid membranes even in the absence of palmitoylation [9] and also in low concentrations of detergent (no lipid bilayer) [10]. An interesting discussion remains - what truly constitutes a GPCR dimer? How close do the receptors have to be to functionally interact? If a dimer is required solely to provide sufficient surface area for G protein coupling, then the proximity provided by such a lipid bridge seems sufficient, but if allosteric regulation by protomers within the dimer is to occur, more direct protein-protein interactions may be required.

These studies remind us that it is vital to consider membrane proteins in the context of their environment. They do not exist in isolation and are heavily influenced by lipids, sterols and other proteins. Cholesterol and palmitoylation can affect all aspects of GPCR activity and their precise roles are likely to be receptor-dependent, although only with more data can we hope to determine any consensus. Membrane environments are challenging to control and probe, but such studies are vital if we are to gain a complete understanding of GPCR function.
